# miR-552 promotes ovarian cancer progression by regulating PTEN pathway

**DOI:** 10.1186/s13048-019-0589-y

**Published:** 2019-12-09

**Authors:** Wenman Zhao, Tao Han, Bao Li, Qianyun Ma, Pinghua Yang, Hengyu Li

**Affiliations:** 1 Department of General surgery, Cao county people’s hospital, East of Qinghe Road, Heze, 274400 Shandong province China; 2 Department of Oncology, General Hospital of Northern Theater Command, Shenyang, 110016 Liaoning Province China; 3 Department of Urology surgery, First Affiliated Hospital of Second Military Medical University, Shanghai, 200433 China; 4 Department of Biliary Tract Surgery, Third Affiliated Hospital of Second Military Medical University, Shanghai, 200438 China; 5 Department of Breast and Thyroid surgery, First Affiliated Hospital of Second Military Medical University, Shanghai, 200433 China

**Keywords:** Ovarian cancer, miR-552, PTEN, Proliferation, Metastasis

## Abstract

**Background:**

Increasing researches have demonstrated the critical functions of MicroRNAs (miRNAs) in the progression of malignant tumors, including ovarian cancer. It was reported that miR-552 was an important oncogene in both breast cancer and colorectal cancer. However, the role of miR-552 in ovarian cancer (OC) remains to be elucidated.

**Methods:**

RT-PCR and western blot analysis were used to detect the expression of miR-552 and PTEN. The impact of miR-552 on ovarian cancer proliferation and metastasis was investigated in vitro. The prognostic value of miR-552 was evaluated using the online bioinformatics tool Kaplan-Meier plotter.

**Results:**

In the present study, we for first found that miR-552 was upregulated in ovarian cancer, especially in metastatic and recurrence ovarian cancer. Forced miR-552 expression promotes the growth and metastasis of ovarian cancer cells. Consistently, miR-552 interference inhibits the proliferation and metastasis of ovarian cancer cells. Mechanically, bioinformatics and luciferase reporter analysis identified Phosphatase and tension homolog (PTEN) as a direct target of miR-552. miR-552 downregulated the PTEN mRNA and protein expression in ovarian cancer cells. Furthermore, the PTEN siRNA abolishes the discrepancy of growth and metastasis capacity between miR-552 mimic ovarian cells and control cells. More importantly, upregulation of miR-552 predicts the poor prognosis of ovarian cancer patients.

**Conclusion:**

Our findings revealed that miR-552 could promote ovarian cancer cells progression by targeting PTEN signaling and might therefore be useful to predict patient prognosis.

## Introduction

Ovarian cancer is one of the most deadly gynecological malignances in the world, causing more than 140,000 deaths yearly [1, 2]. Early detection of ovarian cancer patients with a five-year survival rate is about 90% [3]. However, about 70% ovarian cancer patients were diagnosis at advanced stage and have a poor prognosis [4]. Although as the surgery, chemotherapy and radiotherapy progress, the overall ovarian cancer survival rate has not improved in the past decades [5, 6]. Therefore, it’s urgent to research the underlying mechanism of ovarian cancer and find the new therapeutic target to improving the clinical outcome of patients suffering from ovarian cancer.

MicroRNAs (miRNAs) are a class small non-coding RNA molecule, containing about 22 nucleotides, found in plants, animals and some viruses, which are capable of regulating gene expression at both the transcriptional and translational levels miRNAs [7, 8]. Accumulating evidence indicates that miRNAs play important functions in various biological processes, including cell proliferation, apoptosis, differentiation and migration [9, 10]. More researches also find that miRNAs modulate the proliferation, apoptosis, metastasis and metabolism of various cancers, including breast cancer, lung cancer, liver cancer and ovarian cancer [11–13]. These previous studies remind us that miRNAs may also serve as potential prognosis biomarkers and a novel therapeutic target in various cancers [14, 15].

miR-552 is a newly discovered miRNA, its function and mechanism of action in biological processes and diseases are not completely understood. Previous studies showed that miR-552 promotes colorectal cancer cells proliferation and migration by directly targeting DACH1 via the Wnt/β-catenin signaling pathway [16]. Moreover, miR-552 also enhances metastatic capacity of colorectal cancer cells by targeting a disintegrin and metalloprotease 28 [17]. However, the role of miR-552 in ovarian cancer was little known.

The results of the present study demonstrate that miR-552 was upregulated in ovarian cancer, and that elevated expression of miR-552 was correlated with poor patient prognosis. In addition, we found that miR-552, acting as an oncogene, was involved in ovarian cancer progression, and that miR-552 dramatically facilitated ovarian cancer cell proliferation and metastasis. Notably, it was demonstrated that PTEN was a direct target of miR-552, the levels of which decreased when miR-552 was overexpressed. Our results highlight the importance of miR-552 in promoting the proliferation and metastasis of ovarian cancer cells.

## Materials and methods

### Tissue samples

In total, 80 cases of tumor tissues and their corresponding adjacent noncancerous tissues were obtained from ovarian cancer patients in Cao county people’s hospital from 2012 to 2017, detailed clinicopathological features of the patients is described in online Additional file 1: Table S1. Fifteen cases of tumor tissues and their metastases tissues were obtained from ovarian cancer patients in Cao county people’s hospital from 2013 to 2016. Fifteen cases of tumor tissues and their recurrence tumor tissues were obtained from ovarian cancer patients in Cao county people’s hospital from October 2014 to March 2016. Human ovarian cancer and adjacent normal tissues were immediately snap-frozen in liquid nitrogen and stored at − 80 °C. All of the patients provided signed informed consent. The medical ethics committee of Cao county people’s hospital approved the retrieval method for cancer specimens.

### Cell lines and cell culture

HO8910 and HGSOC cells were purchased from Chinese Academy of Sciences, Shanghai, China. The ovarian cancer cells were cultured in RPMI-1640 medium supplemented with 10% fetal calf serum (FCS; Invitrogen, Carlsbad, CA, USA). The cultures were dissociated with 0.5% trypsin and transferred to new six-well plates biweekly. The lenti-vector expressing miR-552 sponge or miR-552 mimic and their control virus were purchased from Shanghai GenePharma (Shanghai, China). LV3-has-miR-552 mimics (5′- AACAGGTGACTGGTTAGACAA − 3′) can mimic high levels of endogenous mature miR-552 in cells. The miR-552 sequence +loop+ reverse complementation (incomplete complementation) was inserted into the vector to express shRNA, which was then cut into mature miR-552. LV3-has-miR-552 sponge (5′- TTGTCTAACCAGTCACCTGTT − 3′) is that the complete reverse complementary sequence (inhibitor) of miRNA, linked by linker, which adsorbs the binding miRNA (it may adsorb but not degrade, or it may lead to degradation of miRNA), resulting in miRNA not functioning biologically. LV3-has-miR-552 mimics and LV3-has-miR-552 sponge were packaged as a lentivirus. The lentivirus titers are about 1 × 10^8^ TU/ml. HO8910 and HGSOC cells were seeded into 6-well plate and then added 100 μl lentivirus. The stable infectants were screening by using puromycin [18].

HO8910 and HGSOC cells were seeded into a six-well plate until they reached 60–70% confluence. Transfection of si-PTEN or its negative control was performed in each well in the absence of serum with siRNA transfection reagent according to the manufacturer’s instructions (Polyplus, Illkirch, France). The sequence of si-PTEN is as follows: 5′- CCACAGCUAGAACUUAUCAAATT − 3′. This siRNA interrupt PTEN transcript variant 1and transcript variant 2. The siRNA was purchased from Shanghai GenePharma (Shanghai, China).

### Cell proliferation assays

For cell proliferation analysis, ovarian cancer cells were seeded in 96-well plates (3 × 10^3^ cells per well). ATP activity was measured using a Cell Counting Kit-8 at indicated time points. ATP activity was measured using a Cell Counting Kit-8 at indicated time points. The procedure was as follows: The cell suspension (100 μl/well) was inoculated in a 96-well plate, and the plate was pre-incubated in a humidified incubator at 37 °C for 1 h. This was followed by the addition of 10 μl of the CCK-8 solution to each well of the plate, and incubation of the plate for 1 h in the incubator. Finally, the absorbance was measured at 450 nm using a microplate reader (Synergy H1; BioTek Instruments, Inc., Winooski, VT, USA) [19].

### Colony formation assay

For colony formation assay, ovarian cancer cells were cultured in 12-well plates (3 × 10^3^ cells/well). The cells were incubated at 37 °C for 7 days and then fixed with with 10% neutral formalin for > 4 h. The cells were dyed with crystal violet (Beyotime, Haimen, China). The cells were photographed under a microscope (Olympus, Tokyo, Japan).

### EdU immunofluorescence staining

For cell EdU immunofluorescence staining, ovarian cancer cells were seeded into 96-well plates and performed using the EdU Kit (RiboBio). The results were quantified with a Zeiss axiophot photomicroscope (Carl Zeiss) and Image-Pro plus 6.0 software.

### Cell migration assays

For cell migration experiments, 2 × 10^5^ ovarian cancer cells were seeded into the upper chamber of a polycarbonate transwell in serum-free RPMI-1640 medium. The lower chamber was added with RPMI-1640 medium containing 20% FBS as chemoattractant. The cells were incubating for 12 h and the chamber was fixed with 10% neutral formalin for > 4 h. The cells were dyed with crystal violet (Beyotime). The cells were then counted under a microscope (Olympus) and the cell number is expressed as the average number of the cells in each field.

### Cell invasion assays

For cell invasion experiments, 2 × 10^5^ ovarian cells were seeded into the upper chamber of a polycarbonate transwell in serum-free RPMI-1640 medium. The lower chamber was added with RPMI-1640 medium containing 20% FBS as chemoattractant. The cells were incubating for 24 h and the chamber was fixed with 10% neutral formalin for > 4 h. The cells were dyed with crystal violet (Beyotime). The cells were then counted under a microscope (Olympus) and the cell number is expressed as the average number of the cells in each field.

### Real-time PCR

For detection of mature miR-552, total RNA was subjected to reverse transcription using a TaqMan MicroRNA Reverse Transcription Kit (Applied Biosystems). qRT-PCR analysis of miR-552 expression was carried out using TaqMan MicroRNA assay kits (Applied Biosystems). Results were normalized to U6 snRNA using the comparative threshold cycle (Ct) method. The miR-552 primer sequences were forward: 5′ AACAGGTGACTGGTTAGACAA 3′, U6 primer sequences were forward: 5′ ATTGGAACGATACAGAGAAGATT 3′.

The total cells RNA were extracted by using Trizol reagent (Invitrogen, 15596–018). Total cDNAs were synthesized by ThermoScript TM RT-PCR system (Invitrogen, 11146–057). The total mRNA amount presented in the cells was measured by RT-PCR using the ABI PRISM 7300 sequence detector (Applied Biosystems). The PTEN primer sequences were forward: 5′ TCCCAGACATGACAGCCATC 3′, reverse: 5′ TGCTTTGAATCCAAAAACCTTACT 3′. The β-actin was used as reference for relative expression calculation and its primer sequences were forward: 5′ GGCCCAGAATGCAGTTCGCCTT 3′, reverse: 5′ AATGGCACCCTGCTCACGCA 3′.

### Western blotting assays

Thirty micrograms of proteins were subjected to sodium dodecyl sulfate polyacrylamide gel electrophoresis and then transferred to the nitrocellulose membrane. The membrane was blocked with 5% non-fat milk and incubated with the primary antibody for 1.5 h. The protein band, specifically bound to the primary antibody, was detected using an IRDye 800CW-conjugated secondary antibody and LI-COR imaging system (LI-COR Biosciences). The primary antibodies were PTEN (1:1000; # 9188, Cell Signaling Technology) and GAPDH (1:5000; #5174, Cell Signaling Technology).

### Luciferase reporter assays

The luciferase assays were carried out using the Dual-luciferase Reporter Assay System (Promega, Madison, WI, USA). Briefly, cells were co-transfected with miR-552 mimics or miR-control and pMIR-reporter luciferase vector containing a specific sequence of wild-type or mutant PTEN fragment, using siRNA transfection (Invitrogen, NY, USA). Cells were collected and lysed for luciferase detection 48 h after transfection. The relative luciferase activity was normalized against to the Renilla luciferase activity.

### Statistical analysis

All statistical analyses were performed using GraphPad Prism (GraphPad Software, Inc. La Jolla, USA). Statistical analysis was carried out using t test or Bonferroni Multiple Comparisons Test: *p < 0.05. A *p* value of less than 0.05 was considered statistically significant.

## Results

### Increased miR-552 expression in ovarian cancer tissues

**Fig. 1:**
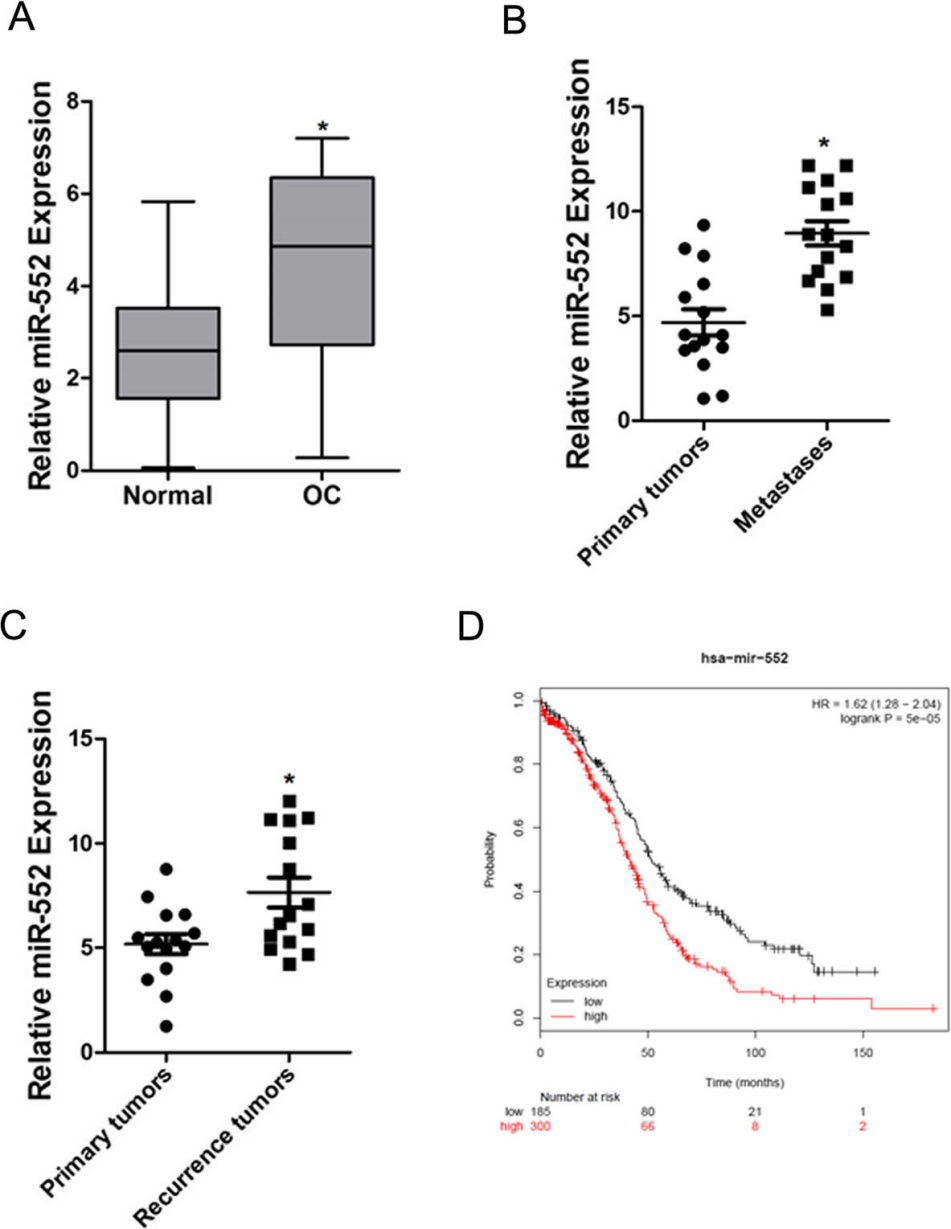
Expression of miR-552 in human OC tissues. **a**. The expression of miR-552 in 80 pairs of ovarian cancer (T) and peri-normal tissues (N) was investigated via real-time PCR analysis. (*p* < 0.05). **b**. The expression of miR-552 in 15 pairs of ovarian cancer (T) and metastasis tissues was investigated via real-time PCR analysis. (*p* < 0.05). **c**. The expression of miR-552 in 15 pairs of ovarian cancer (T) and recurrence tissues was investigated via real-time PCR analysis. (*p* < 0.05). **d**. Kaplan-Meier survival curves of OS based on miR-552 expression in ovarian cancer using the online bioinformatics tool Kaplan-Meier plotter

To explore the role of miR-552 in ovarian cancer progression, we measured the expression of miR-552 in a large set of human OC tissues. As shown in Fig. 1a, miR-552 expression was markedly elevated in OC tissues compared to paired non-tumorous tissues. We also examined miR-552 in metastasis and recurrence OC tissues, which showed that miR-552 expression was notably increased in metastasis and recurrence OC tissues (Fig. 1b and c). We further sought to determine whether upregulation of miR-552 was associated with OC patients’ prognosis. Using the online bioinformatics tool Kaplan-Meier plotter [20], we found that patients with increased miR-552 expression had worse overall survival (OS) (Fig. 1d).

### miR-552 depletion inhibits ovarian cancer cells proliferation

**Fig. 2:**
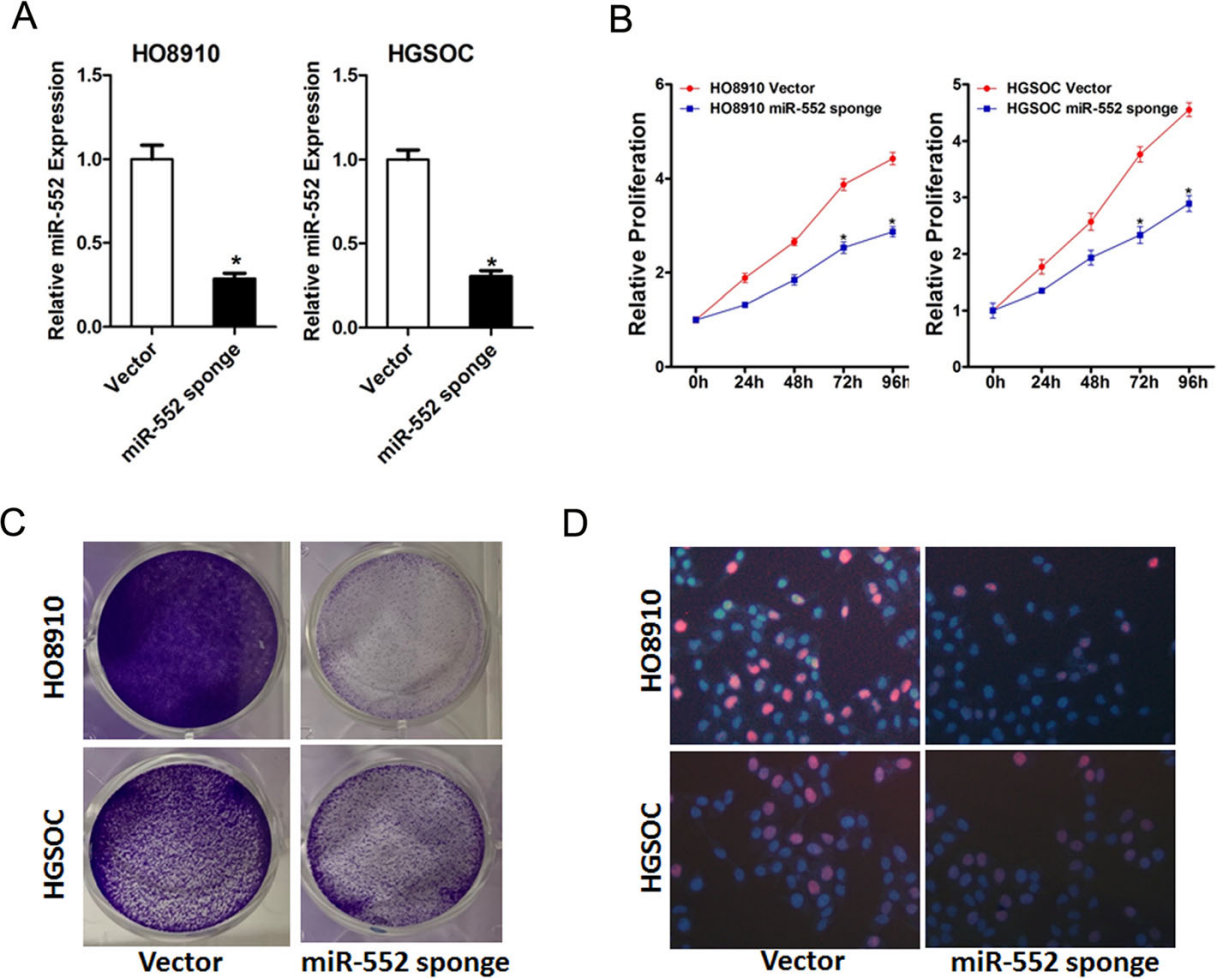
Interference of miR-552 suppresses ovarian cancer cells proliferation in vitro. **a**. The level of miR-552 in miR-552 stably silenced HO8910 and HGSOC cells. **b**. Cell proliferation was measured using CCK-8 assays in HO8910 and HGSOC cells with stable depletion of miR-552. **c**. Colony formation assays of ovarian cancer cells with stable miR-552 sponge. **d**. Cell proliferation was assessed using EdU immunofluorescence staining in HO8910 and HGSOC cells with stable interference of miR-552

To elucidate the effect of miR-552 on ovarian cancer cells behavior, HO8910 and HGSOC cells were infected by miR-552 sponge and stable infectants were established (Fig. 2a). As shown in Fig. 2b, miR-552 depletion repaired the proliferation of ovarian cancer cells markedly. In addition, ovarian cancer cells stably interfered with miR-552 sponge to form fewer and smaller colonies compared with control cells (Fig. 2c). Consistently, 5-ethynyl-2′-deoxyuridine (EdU) staining confirmed that miR-552 knockdown also inhibited ovarian cancer cells growth (Fig. 2d).

### miR-552 overexpression promotes ovarian cancer cells proliferation

**Fig. 3:**
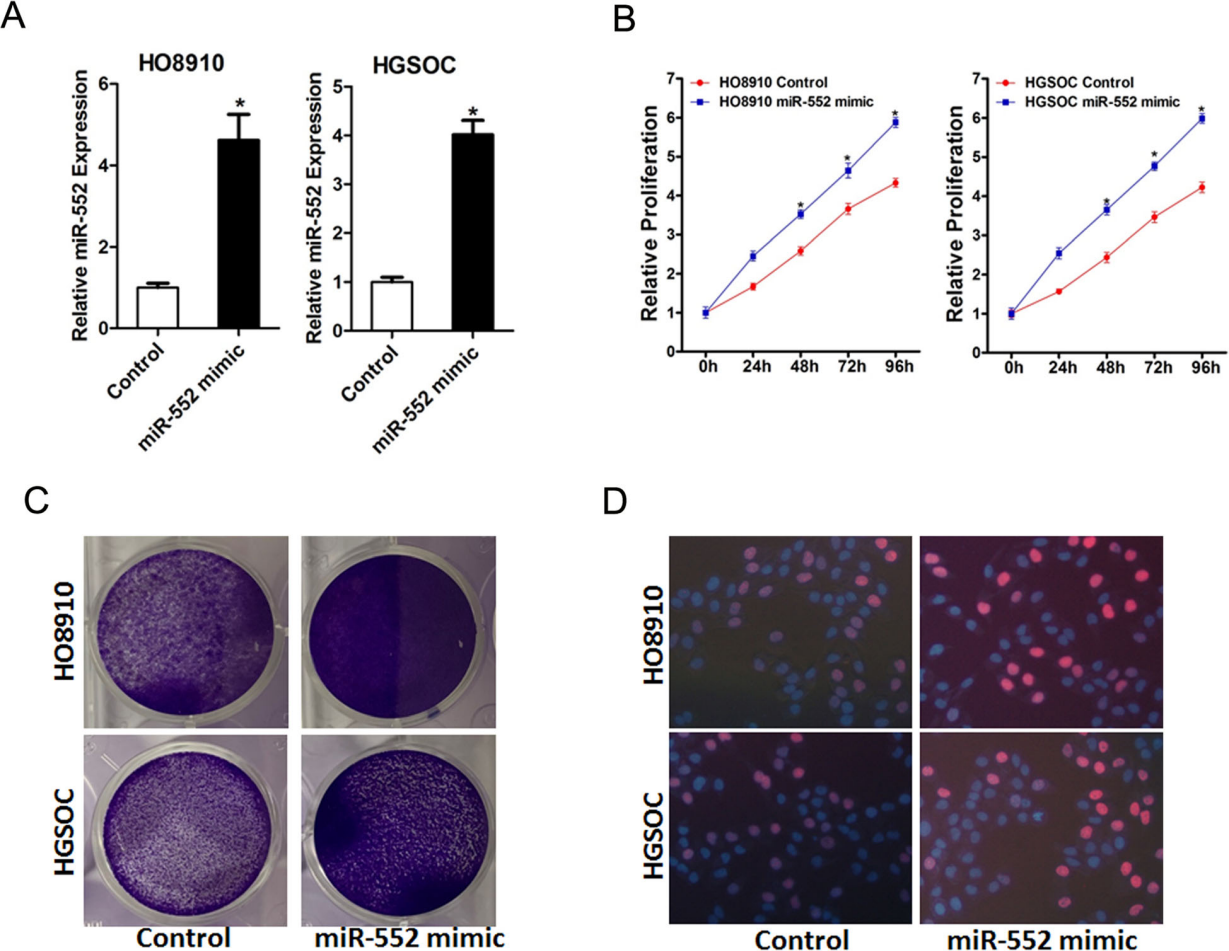
Overexpression of miR-552 facilitates ovarian cancer cells proliferation in vitro*.* **a**. The levels of miR-552 in miR-552 stably overexpressing HO8910 and HGSOC cells. **b**. Cell proliferations were measured using CCK-8 assays in HO8910 and HGSOC cells with stable of miR-552 mimic. **c**. Colony formation assays of HO8910 and HGSOC cells stably overexpressing miR-552. **d**. Cell proliferations were assessed using EdU immunofluorescence staining in HO8910 and HGSOC cells with stable of miR-552 mimic

To further confirm the effect of miR-552 on ovarian cancer cells proliferation, HO8910 and HGSOC cells were infected by miR-552 mimic and stable infectants were established (Fig. 3a). As shown in Fig. 3b, miR-552 overexpression dramatically enhanced the proliferation of ovarian cancer cells. In addition, HO8910 and HGSOC cells stably overexpressing miR-552 formed more and bigger colonies compared with their control cells (Fig. 3c). Consistently, EdU staining also confirmed that ectopic expression of miR-552 inhibited ovarian cancer cells growth (Fig. 3d). Taken together, the above data showed that miR-552 promoted ovarian cancer cells growth.

### miR-552 knockdown inhibits ovarian cancer cells metastasis in vitro

**Fig. 4:**
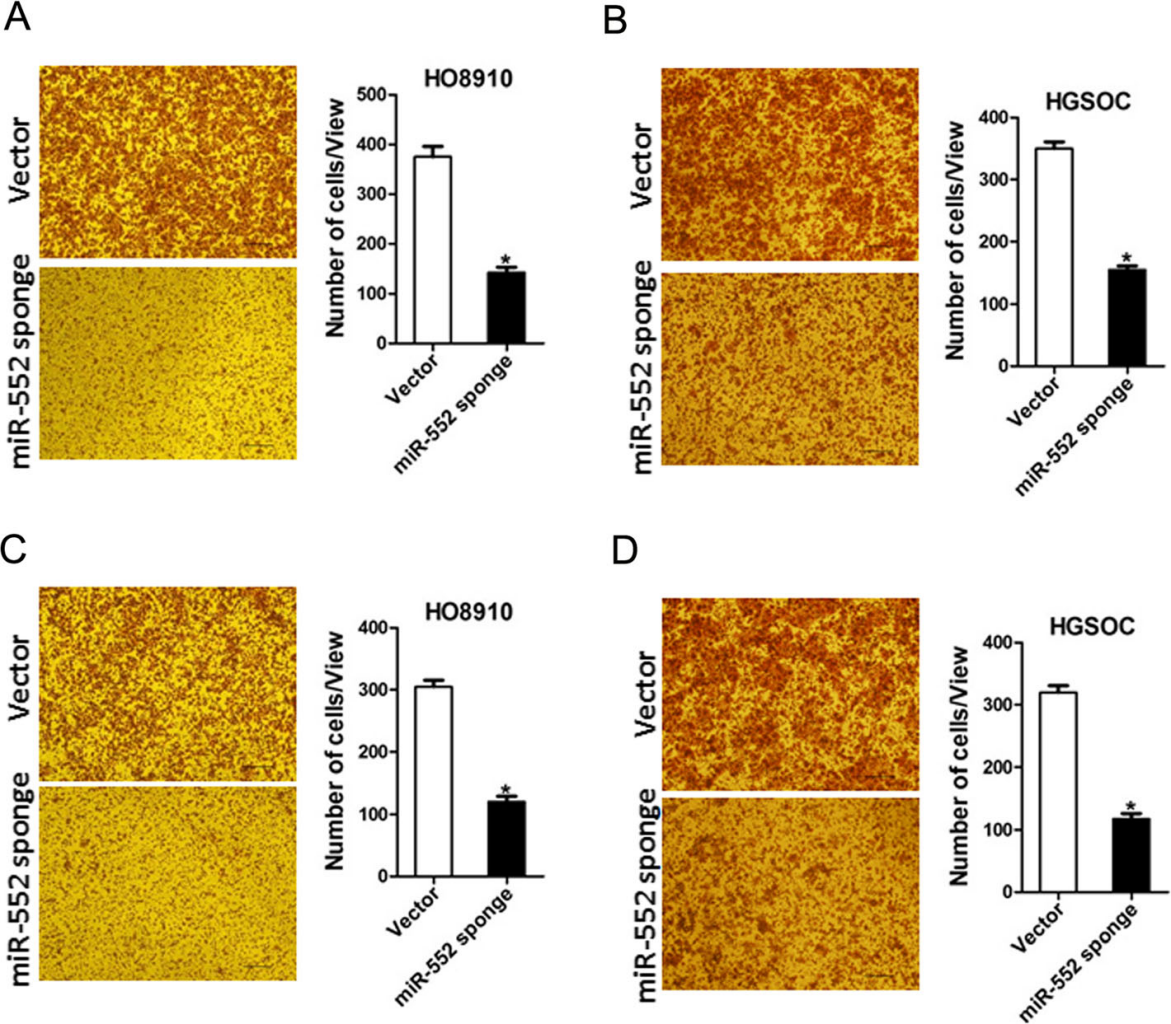
miR-552 depletion suppresses ovarian cancer cells migration and invasion. **a**. The migration ability of HO8910 miR-552 sponge and its control cells was performed utilizing polycarbonate membrane inserts in a 24-well plate. **b**. The migration ability of HGSOC miR-552 sponge and its control cells was performed utilizing polycarbonate membrane inserts in a 24-well plate. **c**. The invasive capacity of HO8910 miR-552 sponge and its control cells were analyzed using Matrigel-coated Boyden chamber. **d**. The invasive ability of HGSOC miR-552 sponge and its control cells was analyzed using Matrigel-coated Boyden chamber

To explore the role of miR-552 in ovarian cancer cells metastasis, transwell assay was performed, showing that the migration ability was impaired in miR-552 inferencing ovarian cancer cells (Fig. 4a and b). In addition, matrigel invasion chamber assay revealed that miR-552 knockdown attenuated the invasiveness of ovarian cancer cells (Fig. 4c and d).

### miR-552 overexpression drives ovarian cancer cell transwell and invasion in vitro

**Fig. 5:**
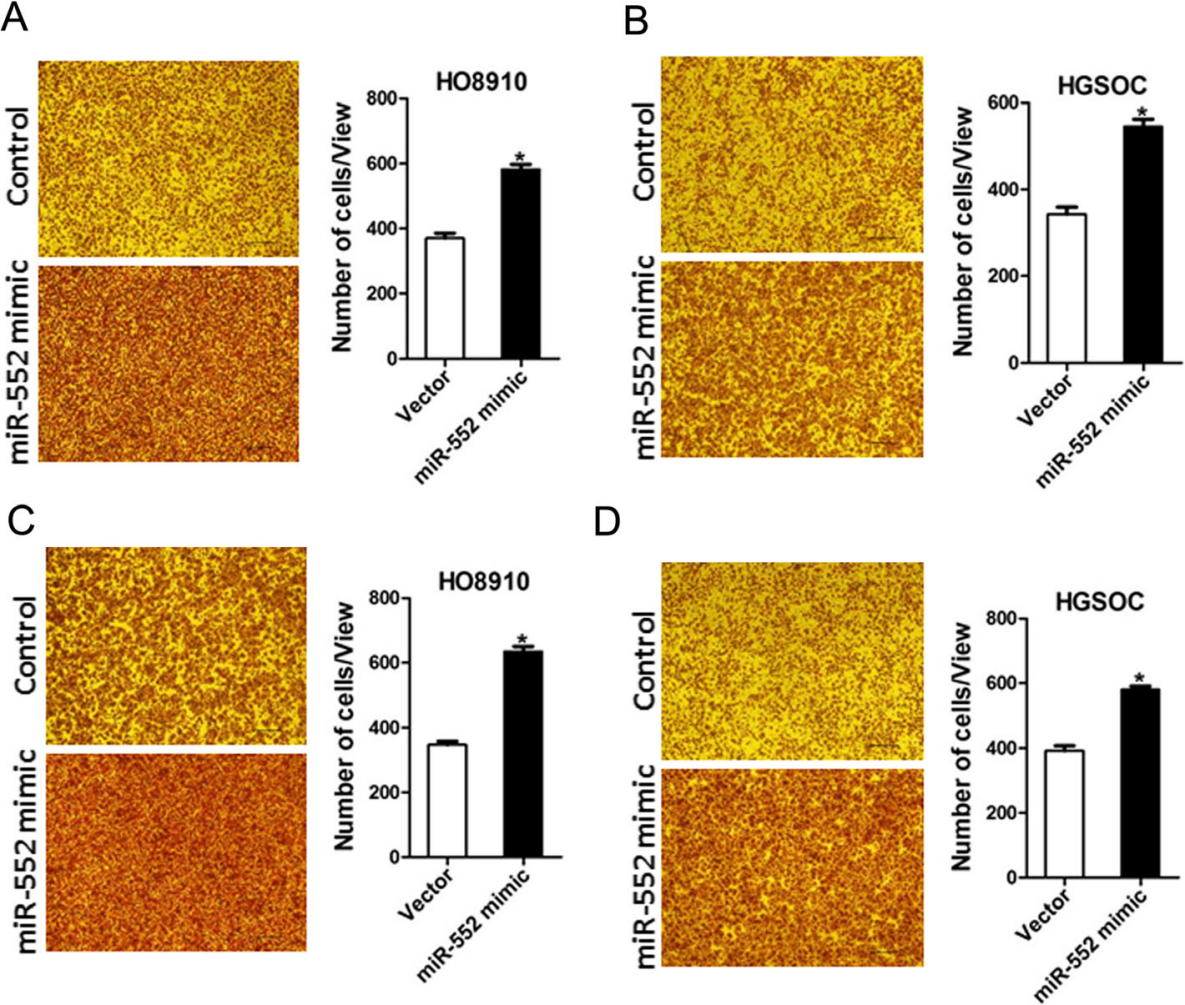
miR-552 overexpression facilitates ovarian cancer cells migration and invasion. **a**. The migration ability of HO8910 miR-552 mimic and its control cells was performed utilizing polycarbonate membrane inserts in a 24-well plate. **b**. The migration ability of HGSOC miR-552 mimic and its control cells was performed utilizing polycarbonate membrane inserts in a 24-well plate. **c**. The invasive capacity of HO8910 miR-552 mimic and its control cells were analyzed using Matrigel-coated Boyden chamber. **d**. The invasive ability of HGSOC miR-552 mimic and its control cells was analyzed using Matrigel-coated Boyden chamber

To further elucidate the role of miR-552 in ovarian cancer cells metastasis, transwell assay was performed, showing that the migration ability was enhanced in ovarian cancer cells overexpressing miR-552 (Fig. 5a and b). Furthermore, matrigel invasion chamber assay revealed that miR-552 overexpression increased the invasiveness of ovarian cancer cells (Fig. 5c and d). Collectively, our results demonstrate that miR-552 disrupted the metastatic potential of ovarian cancer cells.

### miR-552 directly targeted PTEN to promote ovarian cancer cells progression

**Fig. 6:**
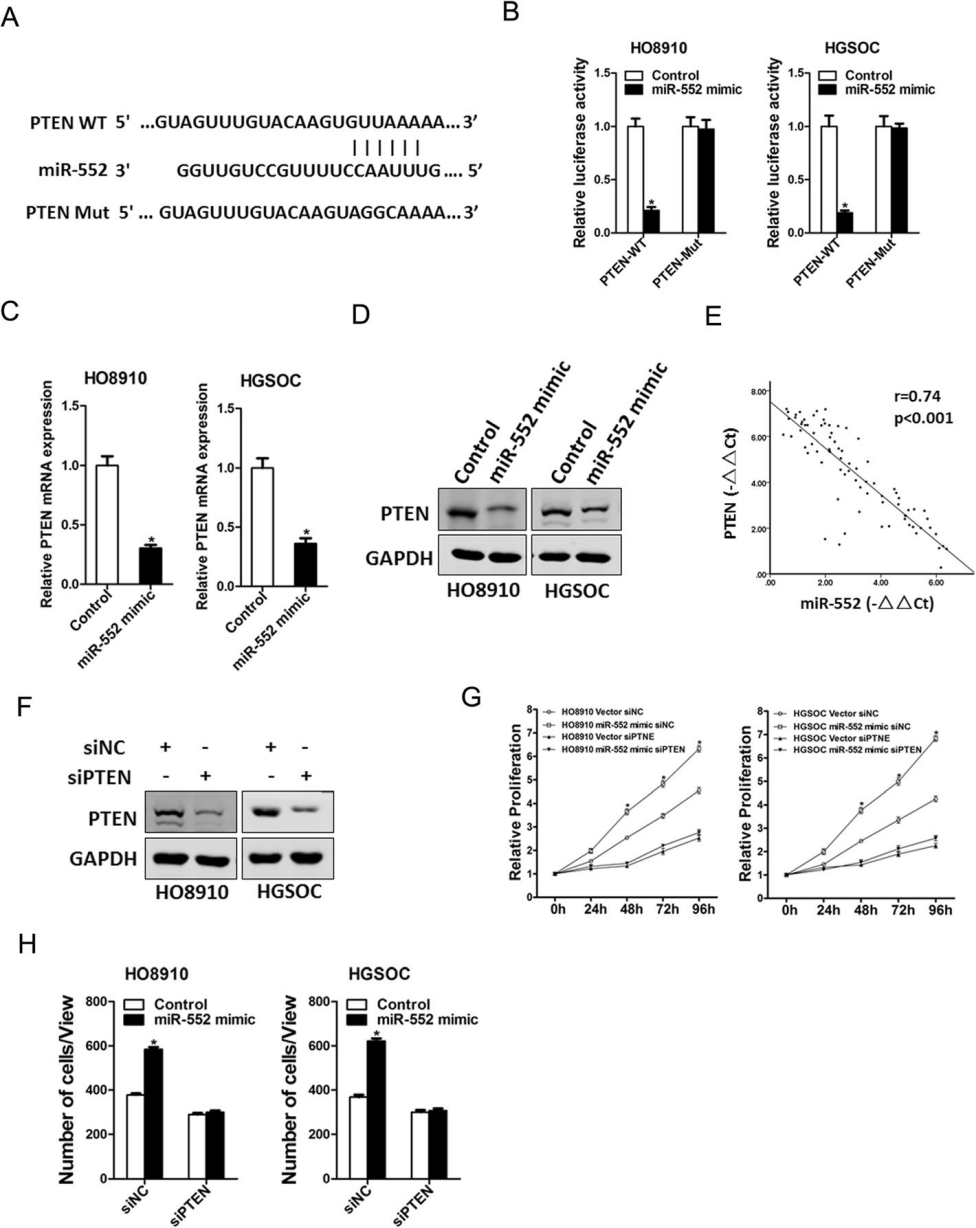
PTEN was a direct target of miR-552 in ovarian cancer cells. **a**. A potential target site for miR-552 in the 3′-UTR of human PTEN mRNA, as predicted by the program Targetscan. To disrupt the interaction between miR-552 and PTEN mRNA, the target site was mutated. **b**. Luciferase reporter assays performed in HO8910 miR-552 mimic or HGSOC miR-552 mimic and their control cells transfected with wild-type or mutant PTEN 3′-UTR constructs. **c**. The mRNA expression of PTEN was checked in HO8910 miR-552 mimic or HGSOC miR-552 mimic and their control cells by real-time PCR. **d**. The protein expression of PTEN was checked in HO8910 miR-552 mimic or HGSOC miR-552 mimic and their control cells by western blot. **e**. Significant correlation was observed between miR-552 and PTEN expression in human OC tissues. **f**. HO8910 and HGSOC cells were transfected with PTEN siRNA and then checked by western bolt assay. **g**. HO8910 miR-552 mimic or HGSOC miR-552 mimic and their control cells were transfected PTEN siRNA and then subjected to CCK8 assay. **h**. HO8910 miR-552 mimic or HGSOC miR-552 mimic and their control cells were transfected PTEN siRNA and then subjected to migration assay

Next, we attempted to identify the target genes of miR-552 that may be involved in ovarian cancer cells expansion. Bioinformatics analysis suggested that PTEN mRNA harbored a putative miR-552 binding site in its 3′-UTR (Fig. 6a). To further explore whether miR-552 directly regulates PTEN expression via interaction with its 3′-UTR, the wild-type or mutant PTEN 3′-UTR reporter plasmids were transfected into miR-552 overexpression ovarian cancer cells and their control cells. The luciferase activity of wild-type reporter was significantly inhibited in the presence of miR-552 (Fig. 6b). However, miR-552-mediated repression of the reporter expression was abolished by mutation of the miR-552 binding site in the PTEN 3′-UTR. Moreover, PTEN mRNA and protein expression was also downregulated in miR-552 overexpression ovarian cancer cells (Fig. 6c and d). There was a significant negative correlation between miR-552 and PTEN mRNA expression in human OC tissues (Fig. 6e).

To investigate the role of PTEN in miR-552-mediated progression of ovarian cancer cells, special PTEN siRNA was used (Fig. 6f). Moreover, PTEN siRNA dramatically attenuated the distinct growth capacity between miR-552 overexpression ovarian cancer cells and control cells (Fig. 6g). Consistently, PTEN siRNA also abolished the discrepancy of metastasis between miR-552 overexpression ovarian cancer cells and their control cells (Fig. 6h), suggesting that miR-552 promoted ovarian cancer cell progression by directly target PTEN pathway.

## Discussion

Ovarian cancer is one of the most common tumors in the female reproductive system and has a poor prognosis, which is related to its complex pathogenesis [21]. Most ovarian cancer patients are diagnosis at late stage and always have distant metastasis [22]. The main treatment used for ovarian cancer patients include surgery, radiation therapy, chemotherapy and targeted therapy or their combination [1]. However, the prognosis of ovarian cancer patients is disillusionary. This was the first study to clarify that miR-552 over-expression correlated well with the poor prognosis of OC patients, suggesting that miR-552 may be a good prognostic biomarker for OC patients.

Increasing evidence has shown that miRNAs are involved in various aspects of carcinogenesis and function as either tumor suppressors or oncogenes [23–25]. The first human disease known to be associated with miRNA deregulation was chronic lymphocytic leukemia [26]. Another role for miRNA in cancers is to use their expression level for prognosis. For instance, low miR-324a levels may serve as an indicator of poor survival in NSCLC samples [27]. Cell-free miRNA are highly stable in blood, are overexpressed in cancer and are quantifiable within the diagnostic laboratory. Circulating miRNAs have the potential to assist clinical decision making and aid interpretation of positron emission tomography combined with computerized tomography [28, 29]. They can be performed at each consultation to assess disease response and detect relapse. miRNAs also have the potential to be used as tools or targets for treatment of different cancers.

It was reported that miRNAs played an important functions in ovarian cancer initiation and progression. For instance, the STAT3-miRNA-92-Wnt signaling pathway regulates spheroid formation and malignant progression in ovarian cancer [30]. miR-6126 is released via exosome and suppresses the progression of ovarian cancer cells [31]. Previous studies showed that miR-552 is upregulated in colorectal cancer tissues and promotes colorectal cancer cells progression by directly targeting DACH1. In addition, miR-552 also enhances metastatic capacity of colorectal cancer cells by targeting a disintegrin and metalloprotease 28. However, the potential role of miR-552 in ovarian cancer has not been reported. In our above work, we found that miR-552 mimic promotes ovarian cancer cells proliferation and metastasis. Consistently, miR-552 sponge inhibits ovarian cancer cells proliferation and metastasis in vitro.

PTEN acts as a famous tumor suppressor gene through the action of its phosphatase protein product [32]. PTEN’s protein phosphatase activity may be involved in the regulation of the cell cycle, preventing cells from growing and dividing too rapidly [33–35]. Mutations of this gene are a step in the development of many cancers, including lung cancer, liver cancer and breast cancer [36, 37]. It also can be used as the targets for drug candidates in tumors [38]. However the exact mechanism beneath PTEN activation in ovarian cancer remains vague. We hereby revealed that miR-552 mimic downregulated PTEN mRNA and protein expression in ovarian cancer cells. Moreover, we also found that miR-552 directly regulates PTEN expression via interaction with its 3′-UTR. More importantly, PTEN siRNA could abolish the distinct growth capacity or metastasis ability between miR-552 mimic ovarian cancer cells and control cells. Herein, we for first revealed that miR-552 promote ovarian cancer proliferation and metastasis via directly regulating PTEN. These findings of the present study not only shed a new light on the mechanism of ovarian cancer but suggest a potential therapeutic target against ovarian cancer patients.

## Conclusion

We demonstrated for the first time that miR-552 expression is upregulated in ovarian cancer tissues, and miR-552 shRNA silencing suppresses the growth and metastasis of ovarian cancer cells. Moreover, miR-552 promoted ovarian cancer cell progression by PTEN signaling pathway. The findings of the present study not only shed new light on the mechanisms responsible for ovarian cancer progression but also suggest that miR-552 may be a novel prognostic marker and a potential therapeutic target for ovarian cancer.

## Supplementary information

**Additional file 1: Table S1.** Clinicopathological features of 80 epithelial ovarian cancer patients**.**

## Data Availability

Data generated from the study are available from the corresponding author on reasonable request.
