# Supplementary material for: miR-552 promotes ovarian cancer progression by regulating PTEN pathway
Source: J Ovarian Res. 2019 Dec 9;12:121. doi: 10.1186/s13048-019-0589-y (PMC6900846; doi:10.1186/s13048-019-0589-y)
Supplement: Supplementary file 1 — Additional file 1: Table S1. Clinicopathological features of 80 epithelial ovarian cancer patients. [file 13048_2019_589_MOESM1_ESM.docx]

**miR-552 Promotes Ovarian Cancer Progression by Regulating PTEN Pathway**

Wenman Zhao ^1, #,*^, Tao Han^2, #^, Bao Li^1, #^, Qianyun Ma^3^, Pinghua Yang^4,*^, Hengyu Li^5,*^

^1^Department of General surgery, Cao county people's hospital, Heze, Shandong province 274400, China

^2^Department of Oncology, General Hospital of Northern Theater Command. Shenyang, Liaoning Province, 110016, China.

^3^Department of Urology surgery, First Affiliated Hospital of Second Military Medical University, Shanghai, 200433, China

^4^Department of Biliary Tract Surgery, Third Affiliated Hospital of Second Military Medical University, Shanghai, 200438, China

^5^Department of Breast and Thyroid surgery, First Affiliated Hospital of Second Military Medical University, Shanghai, 200433, China

^#^These authors contribute equally to this work

**Corresponding authors.** Address: Department of Oncology, Cao county people's hospital, east of Qinghe Road, 274400 Heze, Shandong province, China. E-mail addresses: cxrmyyzwm@126.com (M. Zhao). Department of Biliary Tract Surgery, Third Affiliated Hospital of Second Military Medical University, Shanghai, 200438, China. E-mail addresses: yangpinghua2008@163.com (H. Yang). Department of Breast and Thyroid surgery, First Affiliated Hospital of Second Military Medical University, Shanghai, 200433, China. E-mail addresses: lhy@smmu.edu.cn (Y. Li).

**Running title:** miR-552 drives ovarian cancer cell expansion

**Additional file 1: Table S1. Clinicopathological features of 80 epithelial ovarian cancer patients**

| Characteristics |  |  | Total n=80 |
| --- | --- | --- | --- |
| Age(year) | ≤45 |  | 38 |
|  | >45 | | 42 |
| Lymph node metastasis | Absent | | 50 |
|  | Present | | 30 |
| Distant metastasis | Absent |  | 48 |
|  | Present |  | 32 |
| Histological type | Serous | | 60 |
|  | Non-serous | | 20 |
| Clinical stage | I-II |  | 26 |
|  | III-IV |  | 54 |
